# Notes on the Medical History and Statistics of the British Legion of Spain; Comprising the Results of Gun-Shot Wounds, in Relation to Important Questions in Surgery

**Published:** 1838-10

**Authors:** 


### ART. XI.

*Notes on the Medical History and Statistics of the British Legion of Spain; comprising the Results of Gun-shot Wounds, in relation to important Questions in Surgery.* By RUTHERFORD ALCOCK, K.T.S. &c.; Deputy Inspector General of Hospitals, with the auxiliary Forces in Portugal and Spain.—*London*, 1838. 8vo. pp. 101.

THE opening words of this volume, in which the author sets forth the abundant censure and scant praise,—the toil, peril, and privation,—the hard blows, and still harder words, received in the service of which the medical results are here given to the public, are calculated to disarm the most ruthless critic. We could not have helped receiving the circumstances under which the materials of the work were collected and arranged as an atonement for many imperfections and errors, had such existed: when the book, however, proves to be absolutely a good one, we must consider the credit thence accruing to the writer to be enhanced by those circumstances, which, had they acted on an ordinary mind, would have rendered the work erroneous, confused, and uninstructive.

Had we a hope that any medical work would prove a lesson to governments, we should pronounce Mr. Alcock's likely to answer this purpose; but we can entertain no such hope. How many expeditions have quitted our shores to return thither overwhelmed with disaster, because the page of some well-known medical writer had not been consulted! In the case of the ill-omened expedition which is the subject of the present work, hundreds of living witnesses were accessible who, on the best evidence, could have assured those who planned it that, if the difficulties of the country, climate, and, above all, the government of the Peninsula, were such that they were scarcely surmounted by a well-organized and highly disciplined army, commanded by the greatest general, and supported by the most powerful government and richest treasury in the world, they must prove destructive to a very inferior army, however ably conducted, which received but a *quasi* recognition from British authorities, and was thrown for its supplies on the tender mercies of those of Spain. It is a pleasure to be able to state that Mr. Alcock's work contains indubitable evidence that the superior officers, civil and military, discharged their duty with honour, talent, and energy, and failed only where the very bravest and the very best must have failed.

These remarks may wear the aspect of being extra-professional: they are, however, not so in reality; but have a direct relation to the medical condition of the Legion, so ably depicted by Mr. Alcock. The English and Scotch soldiers of this army were drawn principally from an enervated, oppidan population; whilst the majority of the Irish had been accustomed to spend their days in the open fields, and live more hardly. The effects of this difference in early training was visible in the numbers which these classes respectively furnished to the hospitals at Vitoria; the English being swept in very rapidly, in the proportion of one-third of their number, the Scotch of one-fifth, the Irish but of one-eighth. So long as this miscellaneous and probably ill-organized body remained stationary at Bilbao, the sickness was moderate, and the cases were slight; being such as are ordinarily found in military hospitals in a healthy season. During the march to Vitoria, which commenced on the 29th of October, and terminated on the 3d of December, the troops remained healthy, though the weather became, before it was completed, very severe, as it generally is, during the winter months, in the mountainous district of Biscay, and on the table-land of Old Castile. Sickness assailed them in quarters at Vitoria. The causes are referred to the following heads:—1. An over-crowded state of the town. 2. An inclement winter. 3. Rations bad in quality, deficient in quantity, irregular in delivery, and imperfectly cooked. 4. The state of the hospitals and convalescent depôt generating and aggravating disease, and rendering impossible the adoption of proper means of treatment. 5. Ultimately, deficiency of medical aid.

A portion, at least, of the evils they suffered might have been obviated or removed by good will, exertion, and honesty on the part of the Spanish agents and contractors; but it need not be remarked to those who know anything of Peninsular warfare that of these qualities there was found a total deficiency; whilst their place was supplied by backwardness, malignancy, and treachery, without stint; and that, consequently, the sufferings of the British were frightful. Over-crowding, deficient and bad food, want of blankets or any covering to shield the troops at night from the inclemency of the season, speedily engendered a pestilence, which, of fifty-one medical men, destroyed eleven, and disabled seventeen; out of 7000 troops, sent 5000 to hospital, and carried off a fifth of the force in five months.

The type of fever which committed these ravages is thus described by Mr. Alcock:

"On examining the patient, the medical officer would often find a full pulse, flushed face, dry hot skin, violent pain of the head, a crusted or loaded state of the tongue, with total prostration of strength; at other times greatly diminished vitality, purple and livid countenance, or skin shrunk. Delirium very quickly supervened; the tongue and fauces became dry and hardened, the lips loaded with sordes. Dysentery became developed; the rapid decline of the pulse and all the powers of life followed, and from the tenth to the twentieth day the patient died. During this period the feet, partially or entirely, sometimes including the whole of the legs, would run rapidly through all the stages of gangrene. In others, again, dysentery and its train of symptoms presented the leading feature, with the same fever quickly supervening, running a still more rapid course. If, on the contrary, the patient's state improved, one of his first complaints, on recovering from his delirium, was the tenderness and insupportable sense of heat and pain in the feet; for the relief of which neither bland nor stimulating fomentations, nor friction, nor opiates, general or local, availed. The feet would feel cold to the touch, but present no external appearance of disease or inflammation whatever, and were exquisitely tender: at last, either these symptoms would gradually disappear after a menacing inflammation, or finally separate the tips or the whole of the toes; but, in the worst cases, often involving feet and legs before the line of demarcation indicated its cessation." (p. 20.)

The author says that, if asked to give the chief feature of this fever, he would say "the inflammation and gangrene of the lower extremities." He bestows laudable pains in investigating the causes of this curious symptom, which, if we understand him rightly, occurred under two different sets of circumstances; either in the course of such a fever as has been described, or in emaciated and weakly patients suffering from dysentery, in whom, on their first admission into hospital, the toes of one or both feet were found in a state of gangrene, which was ascribed by the patient to exposure to cold, on some particular occasion specified. When it occurred under the former circumstances, the author referred it "to the near approach to annihilation, in the powers of life continuing at the lowest point consistent with existence for many days, and most fatally experienced at the point furthest from the centre." When the gangrene occurred more suddenly, we should think that there could be little difficulty in ascribing it to frost-bite, (as the patients themselves virtually did;) to which ill-fed, ill-clothed, and weakly persons would be peculiarly prone. We happen to know that this was by no means an unusual occurrence in the mountainous regions in the north of Spain, among the soldiers of the Duke of Wellington's army; especially among those on night-guard or picket. We likewise think it probable that the tendency to gangrene in those affected with fever was connected with the low temperature, from which they were so irregularly and ill protected; for, if the *mere* "approach to annihilation, in the powers of life continuing at the lowest point consistent with existence for many days," could produce it, why is it not observed in the protracted collapse of cholera, which has been known to continue for many days? Again, the author quotes a report from Dr. Neale to Sir James M'Grigor, in which it is stated that, in January 1812, when fever was prevailing in all the general hospitals, the cases at Ciudad Rodrigo had almost universally mortification of the lower extremity, with livor and mortification of the nose. Ciudad Rodrigo is in the north of Portugal; but it consists with our own knowledge that the fever which was prevailing at the same time in the hospitals in the south of Portugal, near the Tagus, was unassociated with any such symptom. The bread given to the men of the Legion was said to have been intentionally mixed with deleterious matter. This the author, like a man of sense, rejects as an absurd rumour; but he describes the bread as having been wretchedly manufactured and of unsound flour; and he asks the very reasonable question, "Knowing, as we do, that diseased rye will produce dry gangrene, why may not some deleterious quality of bad corn produce a similar disease—moist gangrene?" It is not, so far as we know, consistent with medical experience that it does so *directly;* but we have little doubt that the defective nutritious quality of this bad bread was one of the means of inducing that impoverished condition of the system which rendered the soldiers peculiarly liable to the action of cold and other morbific causes.

The treatment adopted consisted in unloading the alimentary canal by an emetic and a purgative; blisters to the nape, to relieve pain of the head; mild diluents, saline effervescents, and gentle diaphoretics; cold immersion and affusion during the hot stage; and, in the latter stages, means suited to support, and even stimulate, the vital powers. The wretched state of the supplies, however, rendered this treatment, simple as it was, always difficult, and sometimes impossible.

A summary of the mortality at Vitoria is furnished by the following table:

*A Return of Admissions, Discharges, and Deaths, from January* 1*st to April* 15*th,* 1837.

| | Febris. | Dysenteria | Varia. | Total. | |
|---|---|---|---|---|---|
| Remained, Jan. 1st | 136 | 5 | 48 | 189 | |
| Admitted............... | 329 | 26 | 163 | 528 | |
| Discharged ........... | 350 | 21 | 152 | 543 | |
| Dead.... ............... | 81 | 9 | 21 | 111 | |
| Remained, April 15th | 44 | 1 | 38 | 83 | Proportion of deaths, 1 in $6\frac{51}{111}$. |

To the mortality stated in the table are to be added 187, who died out of the 787 remaining under treatment, and seventeen deaths during the removal to Santander. When are added to these 400 deaths in December, and as many more at Trevino and other cantonments in the neighbourhood of Vitoria, a total of 1223 is formed. The further additions being made of 500 men incapacitated for future service, and of a mortality of nearly 200 at Santander and Bribiesca, a loss of the services of 2000 men to the Legion (the shocking inhumanity being left out of the question) is to be ascribed to the defective arrangements of the Spanish government or its agents, and the hostility or inertness of the provincial authorities.

Mr. Alcock states the total proportion of mortality in the Legion, during twenty-three months of its service in Spain, thus:—During the first four months, it was about one in forty of the number treated; from the end of the fifth to the ninth month, (the period of its residence in Vitoria,) one in five; and from the tenth to the twenty-third, one in $13\frac{88}{270}$. He is naturally desirous of comparing the mortality of the Legion with that of the British Peninsular army, and finds, from Sir J. M'Grigor's Medical Sketches, and the statements of Mr. Guthrie, that, in the case of regiments composed principally of recruits, which may be considered as forming a fair parallel with the Legion during its disastrous stay at Vitoria, the difference in the loss from sickness is by no means considerable.

The second part of the work is surgical, and contains ample evidence of the professional zeal, method, and activity of the writer. We feel confident, in perusing it, that, in the field and the hospital, he is an excellent military surgeon. Justice, however, compels us to withhold our praise from one section of the book, which has probably cost its author much labour,—the numerical estimates of the results from injuries received in action; most of which are arranged in a tabular form. Some might feel disposed to question the applicability of numbers for any useful purpose to matters affected by circumstances so infinite and minute as this class of injuries. This question we do not feel disposed to argue at present at any considerable length. We agree with the author that, to form a correct average, it should be drawn from a very large number of cases; and think that his statements and tables, correctly calculated, might have constituted a valuable contribution of such an average. On examining them, however, with some care, we find them destitute of that accuracy without which they must be valueless, or worse than valueless. Some of the errors may be typographical; but, even in this case, a list of *errata*, which we have sought for in vain, should have corrected them. Most of them, however, evidently do not belong to this class; and we exceedingly regret that a work, the general execution and tendency of which impresses us very favorably, should be disfigured by them.

There are many parts, however, of the same department of the work, the value of which is much less questionable. Two precepts delivered, as it would appear, by Mr. Guthrie, in his oral lectures, and in the truth of which every skilful military surgeon will concur, receive ample confirmation from the experience of Mr. Alcock. The first is, "Never to be alarmed at hemorrhage, when the finger can reach the bleeding point;" the second, "Be sure to cut the bone short." Mr. Alcock's first operation confirmed the value of both these aphorisms. The very last operation which he performed was, we will not venture to say, unexampled in military surgery, but we must think infinitely rare. The decision in this difficult case was very creditable to his judgment. The humerus was shattered, and the arm was removed at the shoulder-joint. The patient was of a sanguineous and nervous temperament, falling into nervous paroxysms. The divided artery retracted so deeply, that it was impossible to reach it without a second operation for taking up the axillary artery. This was not done; and the stump was lightly dressed, that any further operation might be easily resorted to, should the necessity for it occur. None, however, did occur, for there was no secondary hemorrhage; and the stump healed soundly and rapidly.

The question of primary or secondary amputation, or rather as, following M. Boucher in his Memoir to the French Academy, Mr. Alcock would term it, primary, intermediary, and secondary, is discussed at considerable length. This portion of the work displays less of decision and of reliance on surgical statistics than generally characterize the volume; but it manifests what is of more value, a clear perception of the difficulties of the subject, and a candid statement of the sources whence they arise.

"Unfortunately we are met on the threshold with a great, if not an insurmountable, difficulty. In considering the subject of amputation, we find so many causes influence the result; some, it is true, evident, easy to observe and define; but others difficult to appreciate, although not the less certain in their action: and hence all tables are more or less inconclusive. Thus there are several classes of causes which exercise an important influence over the results of amputation, whether primary or secondary; each branching into a multitude of ramifications, uniting, separating, and again interlacing, bearing upon each other, and finally, by their combination, producing the results upon which alone the reasoning on both questions and the deductions are chiefly drawn." (p. 67.)

After stating his hopelessness of supplying a definitive solution of these questions, but mentioning his intention, in a future work, of furnishing a detailed tabular abstract of between one and two hundred cases; which, though leaving much to be desired, may not prove uninstructive; Mr. Alcock says,

"It is not enough, in my opinion, to say here are two sets of operations, primary and secondary,—(the greater mortality is in the latter, therefore the former is most advantageous;)—a summary mode of settling the question, which would be most desirable, were it just, and as conclusive as the numerical difference is distinct. There are at least certain classes of causes, the influence of which should be correlative and equal in the two opposing sets of operations, before the numerical comparison of results can be final. These I consider to be: 1. The nature of the original injury. 2. The general constitution depending upon the organization and previous health and habits of the patient, and his particular condition at the period referred to. 3. The mode of operation, (in which the time occupied is included,) and a great variety of circumstances, some easily and others with difficulty appreciated or defined. 4. The after treatment, into which the *locale*, the state of the atmosphere, and many other features, enter. 5. The complexion of the mind, and the consequent mental influence which may be beneficial or deleterious to a high degree.

"Unfortunately, a rare combination of favorable circumstances is required to render the observations and registry of a sufficiently large number of facts, thus accurately and fully defined, possible." (p. 68.)

If the question of primary or secondary operations is to remain undecided till their respective merits are tried on two numerous groups of cases, presenting a perfect equality in all the circumstances specified by Mr. Alcock, we need scarcely remark that the decision is adjourned for ever: but we do not consider the authorities in military surgery as having left the question to be settled by Mr. Alcock's impossible experiment. Beside the recorded opinions of Guthrie and Larrey, (they men of experience in the best sense of the term, having combined ample opportunities of observation with the talent required for profiting by it,) it is familiarly known that the practice of primary amputation was that adopted by the surgeons generally in the British Peninsular army, in all cases in which the condition of the patient rendered it admissible, and there was no reasonable prospect of saving the limb. This is not the place for a full disquisition on the grounds of this preference, but the thought would naturally arise in the mind of any one that the repair of a clean incision, to which healthy integuments are carefully adapted, must be a source of much less suffering, and a less serious tax to the patient's constitution, than the sloughing of contused integuments and muscles, and the extensive suppuration occasioned by the presence of comminuted bone, and it may be of foreign bodies; and that, on the first infliction of such an injury, the patient will generally be found in a state more suitable for bearing amputation than after his health and spirits have been broken by confinement, and his constitution has been irritated and drained by the circumstances above mentioned. This plain reasoning appears tolerably conclusive; but still more reliance is to be placed, in a matter of observation and induction, on the universal decision of a body of men whose talent and zeal, and, above all, whose experience, Mr. Alcock, we feel sure, would never think of questioning. The preliminary questions—"Is there a reasonable prospect of saving the limb?" and "Is the shock to the nervous system of the party such, that to inflict immediately the additional one of an amputation would be dangerous?"—it is obvious, demand, in many cases, on the part of the surgeon, a good deal of accurate and prompt thought: in short, of the quality called *tact*, which intelligence and experience alone give, and which no written rules can as yet render medical men independent of.

Mr. Alcock is not opposed to the primary as compared with the secondary operation; but his expressions on this subject are very remote from the decisive tone which pervades the rest of the book: "he hints a fear, and hesitates dislike." The student of military surgery, anxious for information on this point, would rise from a perusal of his volume embarrassed and confused. Some portion of this undecided tone is apparently ascribable to the nature of the force and service with which he was connected. He describes the soldiers of the Legion as in a state of despondency or despair at the thought of primary amputation, preferring even death to sustaining a loss which would reduce them to mendicancy or the workhouse; whilst the experience of weeks of suffering having convinced them, in the case of the secondary operation, of the hopelessness of saving the limb, they submitted with resignation to a loss which they had become convinced was unavoidable. It is obvious that men, certain of being compensated for the privation of a limb by a pension from their country, would have felt differently. There is a degree of fallacy in all reasonings from the class of men composing the Legion to the more energetic soldiers of the regular British army. The author's tables show that, in the case of the former, there was a trifling advantage in secondary as compared with primary operations: the mortality being in the latter as 1 to 2, in the former as 1 to $2\frac{1}{3}$ nearly. In explanation of this he makes the following very judicious remarks:

"As it was only from some accidental circumstance of rare occurrence, after the first action, that cases which, from the nature of the injury, required amputation from the first were deferred, it followed that those who were the subjects of secondary amputation had already proved, by the very fact of their arriving at the second period, that they were among those best capable of bearing the effects of a shock and subsequent disease, and the original injury was generally less severe. It should ever be borne in mind that a cause of fallacy pervades all the returns of secondary amputation given by the advocates of that practice; viz. that the cases are altogether lost sight of which never arrive at the second period, many of which would have been saved by operation at the first, and which, in my opinion, ought strictly to be added to the losses from that practice; an arrangement which would materially affect their tables of results." (p. 98.)

A proportion of the mortality from primary operations was observed to arise from a peculiar febrile condition, which occurred at a period varying from two to three days to ten or twelve after the amputation, and which the author ascribes to the quick succession of two shocks to the nervous system. At least, he found this condition less liable to occur after secondary amputation, when the shocks were necessarily separated by a much longer interval.

Towards the conclusion of the volume there is an abstract of the cases, seventeen in number, of the traumatic tetanus which occurred in the Legion. The first six were treated by bleeding, opiates, and calomel combined with opium; none of the remedies being carried to any great extent: all these cases were fatal. Of the other eleven, six arose from simple flesh-wounds: of these, one recovered, which was treated by carbonate of iron; the others, in which bleeding, acetate of morphia, calomel and opium, and tartar emetic were the remedies, were fatal. The remaining five furnished one case of recovery, though the patient subsequently died of irritative fever with affection of the knee-joint. These cases were treated with opiates, bleeding, and tartarized antimony, all employed to a moderate extent. Amputation after tetanic symptoms had occurred was found, as usual, fruitless. Two cases of recovery out of seventeen is a more favorable result than is generally experienced in this obscure and untractable disease. We regret that a more extensive trial was not given to carbonate of iron, as it was successful in the only case in which it was employed. We have witnessed its failure, but this was only in a single case; whilst the medical officers of the Legion had a comparatively extensive field for trying its powers; and no compunctious visitings need have been felt for withholding the bleeding, mercury, opium, &c., of which the experience of many years has so clearly displayed the inutility.

We have already expressed very strongly our sentiments of the general merit of this work; and we would wish the reader to understand, notwithstanding the character of some of our comments on certain passages, that a minute scrutiny of its contents to the close, has left our first favorable opinion totally unchanged. The military surgeon especially will find it a very valuable, and, what under certain circumstances is by no means immaterial, a very portable addition to his library.

---

